# Metaphors of organizations in patient involvement programs: connections and contradictions

**DOI:** 10.1108/JHOM-07-2020-0292

**Published:** 2021-03-30

**Authors:** Paula Rowland, Carol Fancott, Julia Abelson

**Affiliations:** 1 Occupational Science and Occupational Therapy, Faculty of Medicine, University of Toronto, Toronto, Canada; 2 The Wilson Centre, Toronto, Canada; 3 Canadian Foundation for Healthcare Improvement, Ottawa, Canada; 4 Department of Health Research Methods, Evidence and Impact, McMaster University, Hamilton, Canada

**Keywords:** Canada, Metaphors, Learning, Patient and public involvement

## Abstract

**Purpose:**

In this paper, we contribute to the theorizing of patient involvement in organizational improvement by exploring concepts of “learning from patients” as mechanisms of organizational change. Using the concept of metaphor as a theoretical bridge, we analyse interview data ( *n* = 20) from participants in patient engagement activities from two case study organizations in Ontario, Canada. Inspired by classic organizational scholars, we ask “what is the organization that it might learn from patients?”

**Design/methodology/approach:**

Patient involvement activities are used as part of quality improvement efforts in healthcare organizations worldwide. One fundamental assumption underpinning this activity is the notion that organizations must “learn from patients” in order to enact positive organizational change. Despite this emphasis on learning, there is a paucity of research that theorizes learning or connects concepts of learning to organizational change within the domain of patient involvement.

**Findings:**

Through our analysis, we interpret a range of metaphors of the organization, including organizations as (1) power and politics, (2) systems and (3) narratives. Through these metaphors, we display a range of possibilities for interpreting how organizations might learn from patients and associated implications for organizational change.

**Originality/value:**

This analysis has implications for how the framing of the organization matters for concepts of learning in patient engagement activities and how misalignments might stymie engagement efforts. We argue that the concept and commitment to “learning from patients” would be enriched by further engagement with the sociology of knowledge and critical concepts from theories of organizational learning.

## Background

Described as a shibboleth, a Zeitgeist and an ideology, patient involvement activities have emerged as a mechanism for quality improvement efforts in healthcare organizations worldwide ( Gibson *et al.* , 2012 ; Palmer *et al.* , 2019 ; Madden and Speed, 2017 ). While much of this activity proceeds under twinned banners of moral authority and policy imperatives, social scientists have been building a body of research that explicates and theorizes how these involvement activities work, to what effect and for whom. This process of theorizing is important in any quality improvement activity. It is with this connection to theory that we might best appreciate the impact of quality improvement initiatives, anticipate what can (or should) be scaled up or spread and strategize how these efforts should be progressed ( Davidoff *et al.* , 2015 ). The attention to theorizing may be particularly essential in the domain of patient and public involvement activities, precisely because of the moral imperative of such work. When a stream of activity is accepted as inherently and unquestionably “good”, robust theoretical apparatuses are especially necessary to reveal any unanticipated consequences of these activities or otherwise undetectable “dark sides” of well-intended interventions ( Palmer *et al.* , 2019 ).

One largely unarticulated – but fundamental – assumption underpinning much of patient involvement is the notion that organizations must “learn from patients” in order to enact positive organizational change. Despite this emphasis on learning, there has been a paucity of research that theorizes learning or connects concepts of learning to organizational change within the domain of patient involvement. Where there have been theoretical explorations of learning in these kinds of activities, the focus has been on the ways patients are constructed (or disputed) as legitimate sources of knowledge ( Rowland *et al.* , 2018 ) and how staff members understand and negotiate these constructions of patient knowledge ( Renedo and Marston, 2011 ). Further, these scholarly efforts to connect learning to organizational change have tended to conceptualize organizations as discursive processes, with organizational change understood through dialogical approaches ( Iedema *et al.* , 2010 ).

However, discursive approaches are not the only way to understand or conceptualize organizations or organizational change. Further, while discursive approaches may have the most conceptual resonance with the aims and aspirations of patient involvement programs, it is possible that stakeholders responsible for initiating, supporting, funding and evaluating patient involvement activities may have entirely different understandings of organizations and organizational change. These misalignments have potential consequences for how patient involvement activities proceed, how they are evaluated and what constitutes success. The misalignment may be less about constructions of individuals and their knowledge, but be instead about competing “frames” of the organization ( Goffman, 1974 ). These frames serve as conceptual boundaries to be navigated throughout the engagement work.

In this paper, we contribute to the theorizing of patient involvement in organizational improvement by exploring concepts of “learning from patients” as mechanisms of organizational change. However, rather than attempt to further elaborate concepts and strategies of learning within these activities, we approach the problematic from a different angle. Inspired by classic organizational scholars, we ask “what is the organization that it might learn from patients?” In this way, we seek out a range of conceptualizations of organizations and how these conceptualizations are participating in the conditions of possibility for organizational change. Using the concept of metaphor as a theoretical bridge, we analyse interview data ( *n* = 20) from case study organizations in Ontario, Canada. In the following sections, we will locate our conceptual framing within the use of metaphors in organizational theory. In our analysis, we will explicate a range of metaphors of organizations available within our dataset. Our discussion will explore the potential implications of this range of metaphors and the associated propositions for how we might understand learning from patients as a mechanism of organizational change.

## Metaphors of organizations

Morgan's (2006) classic work on images of organizations serves as a basis for our conceptual framing. In his original book, Morgan categorized various theories of organizations according to their root metaphors. His catalogue of metaphors included organizations as machines, organisms, brains, cultures, political systems, psychic prisons, as flux and change and as instruments of domination. In offering this catalogue of root metaphors, Morgan was not advocating for a particular way of viewing organizations. He readily acknowledged that every view of the organization was necessarily a partial view. Instead, he advocated for the ability to recognize, appreciate and integrate a multiplicity of views while also honing the ability to critically appraise the contributions and limits of dominant metaphors.

Morgan's influential work, elaborated and developed by other organizational scholars, serves as a way to describe and illustrate the wide variance of theoretical perspectives used in study of organizations ( Cornelissen *et al.* , 2008 ). At this level, metaphors are used as sensemaking devices, providing a map through a disparate theoretical field. On another level, the ways metaphors are used in language also provide insight into how individuals make sense of their immediate world. At this level of social interaction, the study of metaphors does more than embellish an existing description of social reality. Here, the study of metaphors provides insight into how individuals construct and shape their world to make it actionable. This approach to metaphors goes beyond considering the use of language within the descriptive domain (i.e. language for understanding) and includes performative uses of language for the purposes of impression management, shaping normative judgements and constructing legitimacy ( Cornelissen *et al.* , 2008 ).

The use of metaphors in organizational research has been the subject of much debate, animating philosophical and methodological arguments in the field ( Boaz *et al.* , 2016 ; Bourgeois and Pinder, 1983 ; Cornelissen, 2005 ; Pinder and Bourgeois, 1982 ; Tsoukas, 1991 ). For the purpose of this research, we engage with the concept of metaphor as a conceptual bridging device. Through our synthesis and interpretation of metaphors of the organization being deployed in the talk and text of patient involvement activities, we seek insight into how individuals are making sense of their organizational context. By extension, this analysis provides insight into how individuals might consider organizational change and the role of patient involvement in actualizing that change. Further, by attending to a range of metaphors in use, we are better situated to understand how some metaphors may reflect dominant understandings of the organizational context while other perspectives are potentially marginalized. These discursive manoeuvres have implications for how processes of learning might proceed, shaping the conditions of possibility for what might be recognized as legitimate and actionable knowledge in the context of organizational change. In summary, we are not engaging with metaphors as literary embellishments or descriptions of organizational life. Nor are we passing judgement on the viability or desirability of particular metaphors. We are analytically engaging with metaphors in order to understand their performative potential and their implications for the aspiration of “learning from patients” as a mechanism of organizational change.

## Methodology and methods

We approached this study as a qualitative discourse analysis based on interviews of individuals involved in patient engagement activities at two healthcare delivery organizations. We had originally designed the study as an interpretive inquiry into the construct of “learning from patients”. Thus, the original intent was an empirical study, asking “what is going on here”? However, following our data collection, we engaged with a more theoretical question of “what is this a case of?” ( Kislov, 2019 ; Tavory and Timmermans, 2014 ), retrospectively adding the analytical layer of metaphorical analysis described below. Interviews were conducted with two groups of individuals: Patient Partners enrolled in organizationally sponsored patient engagement activities ( *n* = 13) and staff members involved in these same patient engagement activities ( *n* = 7). We located this study in two organizations as a means to explore variation but also consistency between the two sites, thereby elaborating our theoretical understanding.

### Description of organizational setting

This study was set within Ontario, Canada. Healthcare in Canada is publicly funded, delivered through provincial and territorial systems and guided by a federal act ( Government of Canada, 1985 ). Publicly funded health delivery organizations are independent institutions governed under provincial corporation acts and operate as non-profit organizations with their own Boards of Directors. All publicly funded health delivery organizations must meet accountabilities determined by the Ministry of Health.

Composed of multiple sites, the first academic health sciences centre in our study offers emergency, acute, long-term and rehabilitative services across more than 1,200 beds. At the time of this study, the organization had been actively developing a robust Patient Partner program with more than 100 Patient Partners enrolled in various engagement activities throughout the health sciences centre. Examples of Patient Partner activities at this organization include single episode quality improvement events, long-standing committee memberships, members of time-limited projects and/or participants in various data collection exercises such as surveys or focus groups.

The secondary study site was a community-based hospital in the same urban centre. This hospital has a long history of community engagement, but the language and strategy around patient engagement is more recent. While there is a range of patient engagement activities currently active in this hospital, our study primarily recruited participants from a single engagement approach: a standing advisory committee of Patient Partners. At the time of this study, the advisory committee has been in place for approximately three years, meets approximately four times per year to provide advice on matters of organizational concern brought forward by staff members and has a membership of approximately 20 advisors. All relevant Research Ethics Boards approved the conduct of this study.

### Procedures

Participants were invited to the study through an email sent by a staff member of the patient engagement programs to an available listserv of all Patient Partners and participating staff members at that site. Participants then approached the study principal investigator with their interest in the study. Potential participants were screened for fit with the study (i.e. ability to conduct the interview in English, ability to provide informed consent and not currently involved in any litigation processes with the organization). All interviews were conducted by the first author between October 2018 and June 2019. The majority of interviews were audio-recorded and conducted as a one-to-one, face-to-face session, lasting between 45 and 90 min. At the request of the participant, one interview was not audio-recorded. In this instance, the participant consented to the interviewer taking detailed notes during the interview. These notes were treated much like field notes, with careful notation practices discerning between paraphrased aspects of the interview and direct quotations from the participant. These notes were included in the dataset. The interview guide was developed iteratively as the study progressed. By the end of the study, the interview was structured along three main areas: (1) a description of the interviewee's patient engagement activities, (2) reflections on the value of patient engagement activities and (3) reflections on the notion of “learning from patients”. All audio-recorded interviews were transcribed verbatim by a professional transcriptionist. Field notes were also collected at the time of each interview. These field notes provided additional context to aid in the analysis.

### Description of interview participants

Of the 13 Patient Partners interviewed, 6 identified as female and 7 identified as male. The average age of the participants was 62.5 (based on 12 responses), with a range from 41 to 83 years. Of the participants, 11 identified as Caucasian. All Patient Partners had post-secondary education, ranging from College ( *n* = 2), Bachelor's ( *n* = 2), Master's degrees ( *n* = 7) and PhDs or other high-level professional degrees ( *n* = 2).

The majority of the staff participants identified as female ( *n* = 6) and one identified as male. While we collected participant information on professional backgrounds and qualification, we are not presenting that information as such details may render the participants identifiable in their own organizations. Average age of staff participants was 45 years, with a range from 36 to 65. All participants identified as Caucasian. Average amount of time working within the organization was 14.6 years (range 6 months–30 years). All participants held leadership positions within the organization, ranging from junior ( *n* = 1) to senior levels ( *n* = 2). All staff participants had a minimum of Master's degrees.

## Analytical strategy

We made use of the qualitative research software HyperResearch^TM^ as a way to collect, organize and sort through various phases of the analytical process. Our analytical process involved multiple iterations of coding, analytical memos and constant comparisons ( Tavory and Timmermans, 2014 ). Our first reading of the transcripts involved a literal reading, looking for conceptual connections within and across transcripts about the various ways participants described themselves and their roles within these patient engagement programs. Our second stage of analysis evolved as the project progressed. We identified the value of metaphors as a second-order concept to be interpreted from the data, using these metaphors as ways to describe and explain the first-order concept we had originally set out to explore ( Cornelissen *et al.* , 2008 ): learning from patients. In this way, our second stage of analysis was discursive. This involved coding specific elements of the transcripts and assigning metaphorical labels to sections of texts ( Cornelissen *et al.* , 2008 ). Where possible, we also noted metaphors that occurred naturally in the talk of the participants. From an analytical point of view, we treated similes as identical to metaphors, as they serve the same interpretive function ( Tsoukas, 1991 ). In this second stage of analysis, we sought as much metaphorical variation as possible.

Having interpreted a candidate list of metaphors from our interview transcripts, we then went back into our dataset to look for systematicity ( Lakoff and Johnson, 2003 ) within the metaphors (i.e. were the metaphors internally coherent) and across the dataset (i.e. were the metaphors taken up, supported, elaborated or contradicted in other interviews; were some metaphors more prevalent in some subsets of the participant group). Throughout our second-order analysis of transcripts and texts, we looked for deviant cases, intentionally searching for evidence that would disrupt our emerging understandings of the metaphors of organizations being put to use. In particular, we looked for differential use of metaphors between staff and Patient Partners. Given the candidate list of metaphors, the research team engaged in discussion and debate about the selection process until we had selected a short list of metaphors that met the following criteria: the metaphors presented have a strong fit with the empirical material, they are plausible interpretations of the extended dataset and they are relevant to a broader intellectual community (as determined through triangulation with existing literature and early presentations of the emerging analysis).

### Reflexivity

Given the interpretive nature of this study, the various standpoints of the research team are relevant to the analytical process. Within our original study team, we had a mix of academics, patient engagement practitioners and various experiences with the health system as patients and/or caregivers. For many of us, those various standpoints are intermingled within one identity. The various standpoints that we each bring to the study – and the effort of moving between standpoints – was part of our conceptual work as a research team throughout the duration of the project. These reflective activities were continued for as long as each individual team member was available to participate in the study. These early reflections shaped the design and early interpretations in the study, however, some team members did step away from the project at a later date as result of their own fluctuating health needs.

## Findings

In this section, we describe various metaphors grouped under three categories: (1) organizations as power and politics, (2) organizations as systems and (3) organizations as narratives. These findings are summarized in Table 1 , outlining the categories of metaphors, examples of individual metaphors and animating ideas associated with each metaphor.

### Organization as power and politics

In this category, we describe metaphors concerned – either implicitly or explicitly – with questions of power and politics. We consider these metaphors political, as they draw attention to decisions that must be made in the context of finite resources. Concepts of competition and alliances circulate here, but the focus of this political activity is split in two directions: internally or externally.

Internally, concepts of power and politics take shape around the notion of *alliances* , where the invocation of “patient perspectives” served to help further a particular stream of strategic activity. The following quotations demonstrate ways in which patient engagement activities – and the perspectives of patients – participate in alliances that afford greater power to the speaker. In the quotations below, the speaker is a staff member, able to utilize what they have heard in a patient engagement activity to facilitate the acceptance of a specific decision or stream of activity.

When I hear feedback from patients, I take that as important. And I've used it. I definitely used it in meetings and said “the patients have said this” and that really shut down a whole conversation. And I, you know, kind of won that battle. (Staff 4)

I think that everyone who works there still would have said “Ok, but I want this, I think this, I think that”. Whereas by saying that this was the recommendation of our Patient Partners, who spent a great deal of time looking at it and considering it, I think that shuts down the debate … people respected that if our Patient Partners that have recommended it … if we are going to say that we are patient-centred, then we need to live by those words. (Staff 5)

To consider organizations as constructed of internal alliances is to bring into the foreground how patient engagement activities are not simply opportunities to “learn from patients” as a matter of neutral knowledge acquisition, but also create support for strategies that may have been otherwise unpopular or difficult to achieve. Thus, this metaphor draws attention to the performative aspects of patient engagement activities and how “learning from patients” can create opportunities to discursively construct alliances with patients, and these alliances work to influence a stream of activity through the active construction of legitimacy based on “this is what our patients told us” (Staff 3). In this political metaphor of organizations, learning from patients could also be framed as patients engaging (or being enrolled) in persuasive work ( Armstrong *et al.* , 2013 ).

In addition to considering how patient engagement activities participate in internal alliances, Patient Partners and Staff brought attention to how these activities participated in external, inter-organizational relationships. Here, there is recognition that healthcare organizations – even in the Canadian context of a publicly funded health care system – externally behave like *competitors* . The two quotations below illustrate how patient engagement programs are seen to be participating in these dynamics of inter-organizational competition.

Well, I think that hospitals, like other organizations, do compete. And I think that one hospital may want to – I do not know this to be true, but I suspect – that hospitals compete at this level too. You know, they do not say “my patient panel is better than yours”, but there is that flavor to some of the things. (Patient Partner 6)

But, I also think there's a lot of pressure within the organization to have patient involvement, but I do not know that that's a universal pressure. Like, I know “Other Hospital” does a great job with patient engagement. Or at least I've heard as such. (Staff 4)

Here, there is an emphasis on how the organization might be positioning itself in relationship to similar organizations, and the processes by which patient engagement programs might contribute to these aspirations. Patient Partner 6 elaborated on this understanding of the organization and the role patient engagement plays in inter-organizational relationships, where

Competition, I think, is an aspect of this. But again, that's something that people in the hospital systems do not or will not talk about. Because it is not good. It's not the image. “We are all helpers here”. … But it is, it's like branding. You know? We want to call it something different, we want to distinguish ourselves. I mean, it might not be a good thing, but it is money. (Patient Partner 6)

Through this attention to inter-organizational competition and the ways patient engagement programs participate in this competition, this framing once again displaces a simple binary between the strategic action of organizations and the lifeworld of patients ( Maguire and Britten, 2018 ). Instead, this Patient Partner seems quite aware of the ways patient engagement programs are participating in strategic action of organizations. Further, in this interview and in others, strategic action is treated as a sensible and understandable mode of organizational change. The notion that organizations compete with one another – and that patient engagement activities might provide an advantage in that competition – is treated as natural.

### Organizations as systems

The second category of interest is *organization as systems* . A less central emphasis on power makes this metaphor distinct from the metaphor of organizations as politics. While there are many different ways that systems can be described (e.g. familial systems, political systems), in this set of metaphors, we bring attention to notions of cybernetic systems and the associated emphasis on networks, processes and feedback ( Von Foerster, 2003 ). In this conceptualization of organizations as systems, there are associated assumptions about the boundaries of these systems. More specifically, the language used by participants suggested implicit (and sometimes explicit) assumptions about who was considered inside the system in contrast to those considered outside of the system.

Consistent with other studies of patient engagement practices, we found participants describing the value of patient engagement work using technocratic rationales ( Martin, 2008 ). In this framing, the organization is conceptualized as a collection of processes, many of which are uncoordinated. There is a strong emphasis on communication systems, with examples of the hospital as a kind of “broken telephone” (Patient Partner 6). Here, the dilemmas of coordination are amplified in academic teaching hospitals, as they are characterized by frequent rotation of students and trainees. While the problems of coordination may be a function of the organization, it is the patient that is perceived to have the most at stake with any failures of communication, a concern articulated in the quotation below:

But you know, at the end of the day, I'm the one that's got (the diagnosis). I cannot leave it here like you can. So, I need the answers. (Patient Partner 13)

In this framing, part of the role of the Patient Partner is to illuminate these communication breakdowns and to help create structures that knit together these broken links. Further, there is a strong sense that a primary purpose of patient engagement activities is to provide feedback, for patients “who have been through all those kinds of dynamics or systems or circumstances to tell (staff) what it is like” (Patient Partner 6). It is with this “real-time feedback” (Staff 5) that systems and processes might be improved. Thus, Patient Partners act much like “process informants” ( Rowland *et al.* , 2018 ), potentially “reaching in” ( O'Hara *et al.* , 2019 ) as a way to augment healthcare systems, particularly as a means to mend broken process links.

On the one hand, Patient Partners were treated as an internal resource within the organization. Here, there was attention to the internal dynamics of patient engagement practices becoming enrolled in the structured systems of approval and relationships that shape the hospitals. For example, Patient Advisory groups became important approval bodies within the organization, where “patient approval” (Staff 7) is achieved through established patient engagement activities of consultation and review. This increased legitimacy within the organizations was discursively treated as a demonstration of the success of patient engagement programs, as these programs became progressively more implicated in the internal operations of the organization.

On the other hand, there was a worry that this increased organizational attention on developing, supporting and legitimizing internal Patient Partner programs was having some unintended effects. In particular, there was a worry that inward focus was achieved at the expense of outward engagement.

So these become the designated patients … I am sure they are worthy people and bring a useful perspective. But if that's the only kind of patient we're going to hear from, or primarily from, then, yes, there might be a silence. We might just paper over the harder conversations. Or we might think we'd validated what we wanted to do anyway. And it's almost like we get a gold star for having heard patients. (Staff 2)

This worry that the organizational attention has tilted towards internal mechanisms of engagement is further reflected in the following quotation: “really, we are not doing that much outside of our patient partners group. So, we are not doing anything really at the community engagement level” (Staff 4).

These boundaries draw attention to a potential irony manifesting in the implementation of patient engagement programs in organizations. This concern appeared more prominently in the interviews with Staff. The concern is that as internal patient engagement programs accrue more resources, develop more support and demonstrate value internally, there is a risk that these programs displace other engagement activities that reach outside the walls of the organization. The language of “community engagement” is being “turned away from” (Staff 7) because of the potential confusion with other developments in the health system. Staff worry that there has been a decrease in forms of “civic engagement” (Staff 2) in favour of newly established patient engagement programs. While it is not necessarily the individuals involved with the patient engagement programs that are reinforcing these boundaries, staff are relaying internalized experiences of gate-keeping where “we considered reaching out to the community, but (the idea) was quickly squashed. Because we have other ways of doing (engagement) now” (Staff 4).

Thus, there was conceptual attention to notion of boundaries. However, those boundaries were primarily addressed in a spatial sense. Patient Partners were considered to be either inside or outside of the organization. In this way, the dynamics of their participation appeared caught in a Catch 22 ( Learmonth *et al.* , 2009 ), where internal participation provided legitimacy but also diminished the potential symbolic capital associated with outsider status ( Locock *et al.* , 2017 ). Further, there was a worry that these Patient Partner programs may be inadvertently rendering the organization less permeable to outside patient and community groups.

### Organizations as narratives

Our final collection of metaphors conceptualize organizations in terms of narratives. In this collection of metaphors, the performative work of language is central to the past, present and imagined future of these organizations. As much as organizations may serve as audiences to Patient Partner stories, they also act as powerful narrators of patients' lives. Many of the interview participants recounted stories of overly gruff, rushed or otherwise insensitive interactions with staff during their course of treatment. The effect of these interactions was often one of feeling vulnerable, belittled or otherwise dehumanized. Individuals returned to the organization as Patient Partners in order to give voice to what could not be said while they were in their patient role. In these acts of giving voice, Patient Partners attempt to shift narratives of patients and patient identities being perpetuated throughout the organization. What is held in common in this set of metaphors is claim that the organization is changed – and is said to have “learned from patients” – only if the internal and external narratives that construct the organization have changed. While this category of metaphors shares an emphasis on the concept of narratives, there was a divide as to whether organizations were conceptualized as (1) inextricably plural collections of narratives or (2) largely coherent (and unified) grand narratives.

Addressing first the notion that organizations are composed of multiple narratives, we first explore the concept of *organization as dialectics* . This metaphor draws attention not just to the presence of conflicting views, but the *necessity* of maintaining and exploring contradictions and tensions. Here, the presence of conflict is not only expected, it is desirable. In the following quote, a Patient Partner describes an interview on a local radio station about a patient engagement program in another hospital. It was that interview that resonated with this Patient Partner and inspired his subsequent volunteerism:

The insight was hearing her interview one day on the CBC and she said “Well, if there is conflict in these meetings and in an organization”, she said, “that's good. If there is a meeting and there is, these things are just rolling along in a hospital and there is no conflict, there is a problem” (Patient Partner 6).

An associated assumption within this metaphor is that the presence of opposing views is necessary for organizational improvement. On one hand, the formality of patient engagement programs may serve to protect these opposing views. For example, one Staff member suggested that the formal oversight of these programs could avoid what she perceived as the potentially problematic practice of clinicians nominating past patients as participants. However, this same Staff member reflected on an experience she found troubling, questioning whether the formal processes of patient engagement programs did entirely protect against patient views becoming “subordinated”:

Sometimes, I've been at a meeting where a patient partner said something that really surprised me. It did not sound very patient-centred. And afterwards, I actually walked out of the meeting with the patient partner and said “That was interesting to me, what you said in that meeting. And I wanted to understand more about that, because it sounded like you changed your point of view, right in the middle of the meeting. Did something prompt that?” And she said “Yeah, I was only thinking of the patient’s point of view. And then I realized that everything is very hard for the staff here. And I need to be more attentive to what they need too”. And so, what I saw happen in the meeting … this patient actually subordinated her interests and needs to those she thought she was hearing around the table. (Staff 2)

In the context of this interview, the staff member was expressing concern about the tendency for even well-intended patient engagement activities to tilt towards organizational concerns, thereby losing the opportunity to learn from opposing views. The salient conceptual feature here is the importance placed on productive opposition as a mechanism of change. In the absence of this kind of opposition, participants may question the value of the engagement activity.

However, despite this emphasis on dialectics, there were also concerns about the potential for Patient Partners to contravene organizational values, creating a new kind of dilemma related to *organizations as aligned and coherent narratives* . As much as there was value placed on the notion of opposition as a productive tension, there was also a declared imperative for all of the efforts (and intentions) to be aligned towards a common aim, unified around the declaration “We try to make things better and we all work together” (Patient Partner 6). However, staff relayed experiences with Patient Partners that were beyond the boundaries of what they could call productive opposition and instead indicated a fundamental divergence from the sanctioned organizational narrative.

One staff member relayed a particularly public experience with a Patient Partner speaking at a group event. Similar stories were recounted in other interviews, suggesting that these stories are taking on the proportions of cautionary tales about how patient engagement activities can “go wrong”. In this particular scenario, Patient Partners had been engaged in a small group meeting to discuss the challenge of encouraging patient adherence to treatment plans. The role of Patient Partners was to help design policy and communication strategies related to policies of treatment adherence. Dissenting opinions and open dialogue were encouraged. However, the following situation created a dilemma.

And we found one Patient Partner who had been assigned to us, who said the most dreadful things about … patients who are not worthy of our care. So, if a patient has a substance use disorder, then they should not be in our hospital. Because “I do not want some dirty, homeless, injection drug user in the bed next to me and my mother”. And I thought, wow, what do I do? That was really tough. (Staff 2)

This potentially creates an identity dilemma: how do hospital representatives meaningfully draw boundaries between valuing dialectics on the one hand (where opposing viewpoints are nurtured and valued), while not tolerating language that is considered offensive on the other? This seemed to be a difficult tension for staff participants to both voice and navigate. Within orientation materials for Patient Partners, there is some language around Patient Partners aligning with organizational “declaration of values”. However, in practice, there are questions about how much the organization can (or should) pre-screen, pre-empt and pre-script Patient Partners to mitigate the risk of breaching those values. To tip too far into such preparation activity may be experienced as silencing the perspectives of Patient Partners and therefore contravening the commitment to dialectics. There was a sense that opposing positions should be valued, but only in so far as those opposing positions aligned with the greater narrative of the organization as expressed through organizationally sanctioned texts such as the mission, vision and declaration of values.

Whereas the first category of metaphors emphasized power and the second category of metaphors emphasized processes, connections and networks, this collection of metaphors brings into focus the social dimensions of organizations, suggesting mechanisms of organizational change through symbolic means (i.e. through shifting symbolic structures and language-games of the organization) ( Morgan, 1980 ). In these metaphors, the organization is taken to be a series of ongoing social constructions, understood through mechanisms of negotiation, accomplishment and enactment. Here, patient engagement activities participate in these ongoing accomplishments by either affirming or disrupting the routine narratives of organizational life.

## Discussion

The novelty of this study lies in explication of metaphorical thinking within the domain of patient engagement activities, particularly as those activities aspire to influence the organization and management of healthcare. In this discussion, we think with, through and beyond these metaphors to consider possible implications for patient engagement programs and their role in shaping healthcare organizations.

### Thinking with the metaphors: implications for learning

In these metaphors, we see that learning is alternately conceptualized as (1) a mechanism for knowledge acquisition and (2) a participatory endeavour of knowledge co-construction ( Sfard, 1998 ). Within the metaphors that view organizations as unified towards a common aim (e.g. organizations as competitors with other organizations, organizations as collections of coordinated processes), there was an emphasis on acquiring knowledge from patients as a means to navigate internal and external politics, to identify and address uncoordinated processes and to move knowledge across boundaries. In contrast, when organizations are viewed as pluralistic (e.g. organizations as dialectics, organizations as internal alliances), there are perceived as being in a constant state of evolution. Here, learning from patients is part of a larger, ongoing construction of organizational knowing ( Cook and Brown, 1999 ) where meaning is constantly being negotiated between various stakeholders.

These alternate understandings of learning have implications for how we might think about the aspiration of patient engagement programs to create generative “knowledge spaces” ( Gibson *et al.* , 2012 , 2017 ; Matthews and Papoulias, 2019 ; Maguire and Britten, 2018 ). In addition to considering these epistemic spaces as characterized by conflicts of value, knowledge and power, there is also the strong possibility of ontological disagreements about the nature of knowledge and associated understandings of learning. If one participant considers the act of learning from patients as a form of knowledge extraction, while another considers learning from patients as an ongoing process of knowledge co-construction, they are likely to have different understandings of the purpose and process of the activity with subsequent implications for the durability of any constructed knowledge space.

### Thinking through the metaphors: implications for organizational change

These metaphors of organizations also have implications for how organizational change might be both understood and recognized in relation to patient engagement programs. Where the organization is understood through metaphors of narratives, advocates (and sceptics) of patient engagement programs may well look for the impacts of patient engagement on organizational narratives. In contrast, where organizations are conceptualized as cybernetic systems, change may manifest in new processes, become codified in new organizational routines or be reflected in new configurations of organizational space. The potential for conflict arises when these metaphors are misaligned, as in the situation where one stakeholder seeks to shift organizational narratives while another seeks more instrumental aims.

Of note in our study, these contrasting metaphors of organizations were not restricted to one stakeholder group or another. By this we mean that we did not find that Patient Partners had more affinity for narrative metaphors while staff members solely deployed metaphors aligned with instrumental aims. This analytical observation provides an elaboration (and potential contrast) to how Maguire and Britten (2018) depict patient and public involvement as liminal knowledge spaces. Using Habermas' division of lifeworlds and systems, Maguire and Britten build an elegant argument for conceptualizing involvement activities as the exchange between the lifeworld of patients and the medical-scientific systems of professionals, clinicians and researchers. In our study, Patient Partners were also using metaphors of organizations that reflect instrumentalist understandings and strategic rationalities. This may be what Maguire and Britten (2018) refer to as “colonization” of the lifeworld, as Patient Partners relayed their experiences using system-based language, concepts and priorities. Alternatively, this may reflect the many complex identities of Patient Partners. Even as Patient Partners are asked to speak to their patient experiences, they do not shed their views of the world that may very well include some well-established metaphors of organizations, including instrumentalist ways of understanding what it means to change an organization.

### Thinking beyond the metaphors: implications of missing metaphors

It is worth noting other prominent organizational metaphors that are conspicuous for their absence in this dataset. For example, we could not find in our dataset any robust reference to the metaphor of organization as brain ( Morgan, 2006 ). While our description of systems has some resonance with how Morgan describes foundational concepts in cybernetics, the connection is relatively fragile. What is missing in our dataset is any vigorous connection to concepts of single or double loop learning ( Argyris, 1997 ), principles of holographic design ( Morgan, 2006 ) or theories of decision making ( Cohen *et al.* , 1972 ). Further while the label of learning organization was used by one participant in one instance, the underlying concepts that animate that particular metaphor were not actively present in that interview or others. This metaphorical absence has implications for the aspiration to connect patient engagement programs to the language of learning organizations ( Fenwick, 1996 ; Eisenberg, 2017 ; Senge, 2006 ; Gherardi, 2009 ) that has permeated many North American hospitals.

Another notable absence in our dataset was the metaphor of organizations as democracies. While organizations were conceptually treated as pluralistic, there was not a strong emphasis within our dataset that would support a more radical ( Burrell and Morgan, 1979 ; Morgan, 2006 ) understanding of organizations. There was no language that would suggest the purpose of patient engagement is to overthrow existing structures in favour of an entirely new organizational form. This absence could certainly be an artefact of our sampling. However, our observation of this absence has implications for patient engagement programs that have implicitly (or explicitly) taken up more radical understandings of engagement anchored in democratic rationales for patient engagement programs and are attempting to implement these change efforts within organizations that are not otherwise viewed as democracies.

### Connecting metaphors, boundaries and learning from patients

Through our analysis, we interpreted several metaphors of organizations circulating within the talk and text of the two case study sites. Throughout these metaphors, there was an underlying assumption about the nature of boundaries. This was predominately a spatial understanding of organizations as having an inside and an outside. This conceptualization of organizations has been fruitful within the patient engagement literature, providing ways to conceptualize patients as maintaining legitimacy largely because of their outsider status ( Locock *et al.* , 2017 ; Martin *et al.* , 2015 ). Further, this spatial conceptualization of patients as outside of the organization provides a conceptual link to notions of learning at and across boundaries ( Akkerman and Bakker, 2011 ), a line of thinking that has been developed in sociocultural and practice based theories of learning ( Lave and Wenger, 1991 ; Engestrom *et al.* , 2007 ).

Without discounting the value of this conceptualization of boundaries, we layer in an alternative interpretation, sensitized to the concept of metaphors. To start the analysis of patient engagement programs with the assumption of patients as outsiders to the organization is to adopt a spatial metaphor. An alternative approach could involve tracing *epistemic* boundaries, considering what metaphors of organizations demonstrate affinity for one another and what metaphors are marginalized. For example, technocratic metaphors of organizations may predominate in patient engagement programs, potentially to the exclusion of more radical metaphors. Patients may identify as outsiders to the organization but have affinity for the dominant metaphors that currently shape the organization. Here, the notion of insider versus outsider becomes more complex, as stratifications of insiders and outsiders become organized by their affinity to dominant organizational metaphors. A shared metaphor may serve a bridge, crossing the insider/outsider divide. Alternatively, dissonant metaphors may serve as an obstacle, further marginalizing discordant voices. This further complicates the notion of a single, unified patient voice positioned outside of the organization ( Rowland *et al.* , 2016 ; Voronka, 2016 ) and instead points towards the ways patients voices are assembled in patient engagement programs.

By connecting concepts of boundaries, metaphors and conceptualizations of learning from patients, we argue against treating boundaries between patients and organizations as a pre-existing map. Rather we seek to draw analytical attention to processes of boundary work ( Abbott, 1995 ; Langley *et al.* , 2019 ; Star, 2010 ). In this way, we maintain focus on the selective permeability of boundaries ( Kislov, 2018 ) in this complex landscape of patient engagement. In considering boundaries in this way, we also include concepts of agency, power and conceptualizations of knowledge. We argue that metaphors-in-use may be a useful entry point into tracing these boundaries and their potential implications.

### Limitations of the study

The value of this interpretive work is in the capacity to extend our collective thinking about patient engagement in organizations. In this way, we are not making any claims about the validity of these metaphors. Nor are we making any claims about the relative value of these metaphors. Further, in contrast to the broader work in organization studies that emphasizes the heuristic value of metaphors to explain organizational behaviour ( Cornelissen, 2005 ; Morgan, 1980 ; Tsoukas, 1991 ), in this study we merely aim to explicate metaphors currently circulating within patient engagement programs. Our aim was to draw attention to the performative work that these metaphors do within the engagement activities and explore the implications of these conceptualizations for the declared aims of patient engagement programs. The kinds of knowledge claims that are possible within each of these metaphors – and the value of these knowledge claims - is a matter for future empirical study ( Tsoukas, 1991 ). Further, we recognize the limits placed on this study by the boundaries of the organizations we chose as empirical sites. These two sites organized their patient engagement practices around concepts familiar to quality improvement strategies ( Armstrong *et al.* , 2013 ). Concepts and practices related to co-design ( Donetto *et al.* , 2015 ; Iedema *et al.* , 2010 ; Williams *et al.* , 2020 ) were not highly visible in the talk or text of participants. Future studies on the metaphors that animate co-design practices would provide a useful and interesting contrast to this study.

## Conclusions

In this study, we sought to develop theoretical knowledge around the notion of learning from patients for the purposes of organizational improvement. In the process, we turned to the use of metaphors, using the concept of metaphor as a way to understand – and make explicit – some of the assumptions that are animating current efforts towards patient engagement at the organizational level. In this paper, we outlined a series of metaphors for consideration. While we argue that each metaphor affords the possibility of distinctive insight into processes of learning within organizational contexts, we recognize that each metaphor is inherently partial ( Morgan, 1980 ). Rather than valorizing a particular metaphor over others, we attend to the wide-ranging pluralism that defines this epistemic space of “learning from patients” at an organizational level.

The value of attending to metaphors is in the opportunity to ask new questions, invite new ideas and to explore conceptual links that might have otherwise remained obscure ( Cornelissen, 2005 ). Through the exploration of metaphors of organizations, we have indirectly asked how the framing of organizations matters for concepts of learning in these patient engagement programs (borrowing a strategy from others, e.g. Elkjaer and Brandi, 2014 ). In the process, we have been able to connect to ideas and concepts with origins in the sociology of knowledge ( Merton, 1937 ), inviting dialogue around concepts of power, knowledge standpoints and knowledge claims and accountability. Further, this exploration of metaphors invites a connection between the concept of “learning from patients for organizational change” to concepts of organizational learning. This makes it possible to explore how competing ideas about learning that permeate the organizational learning literature (e.g. behavioural perspectives, cognitive perspectives and practice-based perspectives on organizational learning synthesized by Elkjaer and Brandi, 2014 ) might intersect with and shape the possibilities for learning from patients in these organizations. These conceptual intersections and their implications also shape what kinds of organizational changes might be deemed possible, or even desirable, thereby having implications for how we might understand the impact of these patient engagement activities.

This exploration of the various metaphors that underlie patient engagement activities may serve both patient engagement practitioners and participants. Reflexivity in practice is important for all stakeholders in patient engagement activities, providing a means to explore assumptions about what it means to “learn from patients” and how that learning is related to organizational change. It is in this kind of unpacking that potentially competing frames of the organization might be revealed. In the absence of revealing these frames, organizations may inadvertently create misalignments between the development of activities, the expectations of participants and the evaluation of impact. These kinds of misalignments have implications for the sustainability the activities but also the collective good will and energy that has been invested by various stakeholders. To avoid these kinds of misalignments, it is wise to have early conversations about what is meant by learning from patients and how such learning is to be related to organizational change.

In conclusion, this thread of analysis serves as a counter-point to what has been labelled as the “flight to empiricism” in patient engagement ( Beresford, 2002 ). This flight to empiricism has been characterized by the copious production of guidelines and “best practices”, where the pace of activity might potentially elide the political and ideological underpinnings of engagement programs ( Gibson *et al.* , 2012 ). We further wonder if this rush to activity might also render invisible the contested nature of knowledge under aspirational statements such as “learning from patients”. If we consider patient engagement programs as participating in creating knowledge spaces, inviting the knowledge of patients to interact and influence the knowledge of organizations, then we must also anticipate that knowledge spaces are messy and complex, likely to be characterized by conflict as well as by exchange ( Nerland, 2018 ; Maguire and Britten, 2018 ). We argue that the concept and commitment to “learning from patients” would be enriched by further engagement with the sociology of knowledge and critical concepts from theories of organizational learning.

## Figures and Tables

**Table 1:** Summary of metaphors

Organizational metaphor	Conceptualization of organizational change	Conceptual relationship to patient engagement activities
Power and politics	Power is a central concept and concern. Organizational change manifests through persuasion and dynamics of competition	Along with other strategies, patient engagement programs participate in the politics of persuasion within the organization
		The presence of a robust patient engagement program potentially bolsters the reputation of the organization
Systems	The organization consists of a series of interrelated processes. Patients may act as “process informants”, providing insights into these processes. Organizational change manifests through the refinements of these processes	Patient engagement programs are considered successful when they are embedded in organizational structures, continually participating in process improvements
		Robust patient engagement programs may direct attention internally, potentially eclipsing outward facing and/or community engagement activities
Narratives	Language and meaning-making are central concerns. Organizational change manifests through the shifting of narrative or the “re-storying” of the organization	Patient engagement programs enrich the organization by providing alternate narratives. These contradictions and tensions are productive
		Patient engagement programs must also fit the “sanctioned narrative” of the organization, particularly as related to the declared values, mission and vision. Divergence from this narrative is experienced as potentially problematic
